# Individual and Task-Specific Variability in Violin-Playing Kinematics Among Professional and Amateur Performers: A Pilot Motion-Capture Study

**DOI:** 10.7759/cureus.116123

**Published:** 2026-09-11

**Authors:** Risa Suzuki, Takahiro Ohkawa, Toshihiko Sato, Yasuhisa Ueda

**Affiliations:** 1 Faculty of Human Sciences, Waseda University, Tokorozawa, JPN; 2 Department of Physical Therapy, Faculty of Health Science Technology, Bunkyo Gakuin University, Fujimino, JPN; 3 Department of Physical Therapy, Faculty of Health Sciences at Narita, International University of Health and Welfare, Narita, JPN

**Keywords:** individual variability, motion capture, musician kinematics, participant-level analysis, performing arts medicine, pilot study, playing-related musculoskeletal disorders, upper-limb kinematics, violin performance

## Abstract

Background

Violin performance requires coordinated multi-joint and postural control, and performers may adopt different movement strategies. This pilot study primarily described participant-level heterogeneity and task-specific kinematic patterns in professional and amateur violinists, with secondary exploratory screening for nonoverlapping group ranges.

Methods

Six violinists (three professional and three amateur) were assessed using a 16-camera Vicon motion-capture system (Vicon Motion Systems Ltd., Oxford, UK) during a standardized phase using the same violin, bow, and shoulder rest. Three tasks with complete data were analyzed: a four-note slurred scale, a spiccato scale, and a concerto excerpt. Ten kinematic outcomes were derived from right upper-limb geometric proxy angles, pelvic-center displacement variability, and wrist path length. Analyses were descriptive and hypothesis-generating. Thirty task × outcome combinations were screened for complete nonoverlap between groups; with three participants per group, approximately three such combinations are expected by chance.

Results

Substantial interindividual heterogeneity was observed within both groups. Three of 30 task × outcome combinations showed nonoverlapping ranges, matching the number expected by chance, and none recurred across tasks. During spiccato, anteroposterior and vertical pelvic-center displacement variability were higher in all professionals; during the concerto excerpt, right wrist proxy-angle temporal variability was lower in all professionals. The 0.15-mm vertical pelvic-center separation was not interpreted as meaningful because study-specific measurement precision was not established.

Conclusions

No kinematic pattern consistently distinguished professional from amateur performers across tasks. The dominant finding was substantial participant-level heterogeneity and task-specific kinematic variation. Larger studies with repeated measurements and prospectively assessed playing-related musculoskeletal symptoms are needed to evaluate the clinical relevance of individualized movement profiles.

## Introduction

Violin performance requires repetitive, coordinated movements of the shoulder, elbow, wrist, and trunk, with performers continuously adjusting multi-joint motion to meet task-specific bowing demands [1,2]. Such prolonged and repetitive upper-limb activity has motivated clinical interest because playing-related musculoskeletal disorders (PRMDs) are common among violinists and other instrumental musicians and can involve pain, functional limitation, and interruption of musical activity [3,4]. At the same time, motion-capture studies indicate that violinists can employ substantially different movement strategies depending on the performer and bowing task [5], while marker-based 3D analysis has also demonstrated clinical feasibility for quantifying upper-body kinematics in violinists and violists [6]. This combination of clinical heterogeneity and performer-specific motor behavior raises the possibility that violin-playing kinematics may be more appropriately characterized at the individual and task levels rather than solely through group-average patterns.

Previous studies have provided important evidence that violin-playing kinematics can differ according to expertise level, including motion-capture approaches designed to distinguish higher- and lower-skill performers [7]. However, expertise-related group comparisons do not necessarily imply that performers within the same expertise category use a uniform movement strategy. In fast repetitive violin bowing, coordination has been shown to vary systematically with performance conditions, while individual performers can exhibit internally consistent strategies despite substantial interindividual variation [8]. Similarly, joint kinematics in violinists vary according to bow stroke, tempo, and string played, with appreciable variability both within and between musicians [5]. Postural behavior in bowed-string performance can also vary substantially across individuals, further indicating that performer-specific movement organization may be obscured when observations are reduced to group-level summaries [9]. Participant-level and task-specific analyses may therefore provide a useful complement to conventional group comparisons, particularly in small exploratory studies in which a group mean can be strongly influenced by the movement strategy of a single performer.

Accordingly, this descriptive pilot study examined upper-limb and postural kinematics in six violinists (three professional and three amateur) during three technically distinct bowing tasks under a standardized instrument setup. The primary aim was to characterize participant-level heterogeneity and task-specific patterns in right shoulder, elbow, and wrist geometric proxy angles, pelvic-center displacement variability, and wrist path length, reflecting the coordinated contributions of multiple upper-limb and postural components to violin performance [1,5]. Because violin-bowing coordination depends on both performer-specific strategies and task demands, participant-level values were retained rather than reduced to group averages [8].

Three exploratory, secondary questions were also addressed: (1) whether the three professional performers shared common kinematic features; (2) whether any kinematic measure showed a descriptively nonoverlapping range between the professional and amateur groups, recognizing that a small number of such patterns is expected by chance with three participants per group (see Materials and Methods); and (3) how any such pattern varied across the three tasks. These secondary analyses were exploratory screens, not tests of a group-level expertise effect.

This study did not measure pain, disability, fatigue, or playing-related musculoskeletal symptoms. PRMDs are referred to above only as long-term motivation for characterizing performer-specific movement; the present design cannot establish any clinical association, and clinical relevance is discussed only as a direction for future work.

## Materials and methods

Study design

This was a single-session, laboratory-based observational pilot study designed to characterize participant-level and task-dependent kinematic patterns during violin performance. Data collection was conducted on December 26, 2023. Six violinists, comprising three professional and three amateur performers, completed a standardized performance protocol during a single motion-capture session. Data collection consisted of two sequential phases. In the first phase, participants performed the prescribed tasks using their own violin and bow; this phase also served as familiarization with the motion-capture environment and as a warm-up before the primary recordings. In the second phase, all participants performed the same tasks using a common violin, bow, and shoulder rest provided by the investigators. The primary analysis was restricted to this standardized second phase and to the three performance tasks for which complete kinematic data were available from all six participants: a four-note slurred scale, a spiccato scale, and a concerto excerpt. The study was designed as descriptive and hypothesis-generating; individual performers, rather than motion-capture frames, movement cycles, or repeated trials, were treated as the units of analysis.

Participants

Six violinists participated in the study: three professional performers (P1-P3) and three amateur performers (A1-A3). Professional status was operationally defined by two criteria: having earned income through professional violin performance and holding a conservatory-level music degree. All three professional participants met both criteria, whereas none of the amateur participants met either criterion. Participant characteristics are summarized in Table 1. The professional performers were 33-41 years old and had 29-36 years of violin-playing experience, whereas the amateur performers were 21-57 years old and had 6-38 years of playing experience. Weekly practice time also varied substantially across participants, particularly within the amateur group. All six participants were included in the primary analysis, which was restricted to the three performance tasks for which complete kinematic data were available from every participant.

**Table 1:** Participant demographics. Professional status was defined by both performance income and conservatory-level training; all professionals met both criteria, and no amateur met either.

ID	Group	Age (years)	Sex	Height (cm)	Weight (kg)	Years playing (years)	Weekly practice (hours)
P1	Professional	41	Male	164.4	61	36	20
P2	Professional	33	Female	160.1	55.95	29	40
P3	Professional	40	Male	169.4	60.65	36	15
A1	Amateur	57	Female	157.8	57.45	21	15
A2	Amateur	50	Female	158	50	38	2
A3	Amateur	21	Male	170.3	52.3	6	5

Experimental procedure

After marker placement and preparation of the motion-capture system, each participant completed the prescribed violin-playing tasks in two sequential phases during the same recording session. In the first phase, participants performed the tasks using their own violin and bow. Shoulder-rest use followed each participant’s usual practice, and participants who normally used a shoulder rest used their own device. This phase also served as familiarization with the laboratory environment, motion-capture setup, and performance protocol, as well as a warm-up before the primary recordings.

Participants then repeated the prescribed tasks using a standardized instrument setup consisting of the same violin, bow, and shoulder rest provided by the investigators for all performers. This second phase constituted the primary recording phase and was the sole source of data for the analyses reported in the present study. The order of the two phases was fixed for all participants, with the familiarization/warm-up phase always preceding the standardized primary recording phase. Within each phase, the scale-based tasks were performed before the concerto excerpt.

Performance tasks

Participants performed four prescribed violin-playing tasks in a fixed order during each recording phase. All tasks were performed to a metronome set at quarter note = 65 beats/min. The tasks comprised, in order, a scale performed in sixteenth notes with one note per bow; a scale performed with four notes under each slur; a scale performed using spiccato bowing; and a short excerpt from Mozart’s Violin Concerto No. 3 in G major, K. 216. Within each recording phase, each task was performed twice. The first performance served as a practice trial, and the second performance was recorded as the analysis trial. Only the second trial was included in the present analysis. Thus, one analyzed trial was available for each participant × task combination included in the primary analysis. Although all four tasks were included in the recording protocol, usable motion-capture data for the one-note-per-bow scale were unavailable for P3. To ensure that the primary descriptive comparison was based on the same set of participants for every task, this task was excluded from the present analysis. The primary analysis therefore included the three tasks with complete kinematic data from all six participants: the four-note slurred scale, the spiccato scale, and the concerto excerpt.

Motion-capture system and marker configuration

3D marker trajectories were recorded using a Vicon 3D optical motion-capture system (Vicon Motion Systems Ltd., Oxford, UK) operated with Vicon Nexus 2.10.3. The capture system comprised 16 optical cameras, including eight MX T20-S cameras and eight Vero v1.3 cameras. Marker trajectories were sampled at 100 Hz. Retroreflective markers with a diameter of 14 mm were used for the upper-body and trunk regions relevant to the present analysis.

Marker placement was based on the Plug-in Gait Full Body marker set (UPA and FRM configuration, version 3.2). Markers were applied by the same investigator for all participants and were not repositioned between the familiarization/warm-up phase and the standardized primary recording phase. An initial static anatomical-pose trial was recorded as part of the calibration procedure. The Plug-in Gait model outputs were not used for the present kinematic analysis. Instead, the reported upper-limb angles were calculated directly from the labeled 3D marker trajectories as simple three-point geometric proxy angles.

The marker trajectories used for the present analysis included the seventh cervical vertebra (C7), right shoulder (RSHO), right upper arm (RUPA), right elbow (RELB), right forearm (RFRM), right radial and ulnar wrist markers (RWRA and RWRB), right finger marker (RFIN), and the left and right anterior superior iliac spine markers (LASI and RASI). These trajectories were subsequently used to derive the upper-limb geometric proxy angles, pelvic-center displacement measures, and wrist path length described below.

Kinematic outcome measures

Kinematic outcomes were calculated from the 3D marker trajectories for each participant and performance task. Because all participants held the bow in the right hand, the right upper limb was selected for the upper-limb kinematic analysis. Three geometric proxy angles were used to characterize the right upper-limb configuration. The right shoulder proxy angle was defined as the angle formed by C7, the right shoulder marker, and the right elbow marker. The right elbow proxy angle was defined as the angle formed by the right shoulder, right elbow, and right wrist positions. The right wrist position was defined as the midpoint between the right radial and ulnar wrist markers (RWRA and RWRB). The right wrist proxy angle was defined as the angle formed by the right elbow, right wrist, and right finger positions. For each proxy angle, the time-series mean and SD were calculated to describe the average limb configuration and its temporal variability, respectively.

Postural motion was characterized using the midpoint between the left and right anterior superior iliac spine markers (LASI and RASI) as an operational estimate of the pelvic center. The SD of pelvic-center position was calculated separately in the mediolateral (X), anteroposterior (Y), and vertical (Z) directions to quantify positional variability during performance. Wrist path length was calculated as the cumulative frame-to-frame displacement of the right wrist position over the analyzed recording.

These upper-limb angles were simple three-point geometric measures and were not intended to represent anatomically rigorous 3D joint angles derived from segment coordinate systems. They were therefore interpreted as proxy measures of upper-limb configuration rather than as clinical joint-angle measurements.

Because wrist path length is a cumulative measure that scales with recording duration, it was also expressed per second of the analyzed recording as a robustness check. This normalization did not change which task × outcome combinations met the descriptive nonoverlap criterion (see Results).

Two senses of “variability” are distinguished throughout this article. Within-trial temporal variability denotes the SD of a kinematic signal over time within a single trial; it is the basis of the proxy-angle SD outcomes and the pelvic-center displacement outcomes. Interindividual heterogeneity denotes the dispersion of participant-level values across performers. The two are not used interchangeably.

Data processing and quality control

3D marker trajectories were extracted from the C3D files using Python with the ezc3d and NumPy libraries. Recordings were classified according to participant, performance task, and recording phase based on the file-naming convention and session records. Only recordings from the standardized primary recording phase were included in the present analysis.

For each participant and task, kinematic outcomes were calculated from the single analysis trial recorded after the practice performance. No repeated analysis trials were available for estimating within-participant trial-to-trial variability. No kinematic outcome values were imputed at the analysis stage.

Data completeness was reviewed before the primary analysis. All six participants had usable data for the four-note slurred scale, spiccato scale, and concerto excerpt under the standardized recording phase. Primary analyses were restricted to the three tasks with complete data across all six participants. The analysis scripts read the source C3D files without modifying the original motion-capture data.

Marker gaps were filled in Vicon Nexus before C3D export. Spline fill was used for gaps within the default maximum gap length of 100 frames. For gaps not suitable for spline filling, including some longer gaps, pattern fill or rigid-body fill was used according to the marker configuration. Based on the operator’s recollection, many gaps were brief, although some exceeded 100 frames. When either the radial or ulnar styloid marker was missing, the other wrist marker on the same wrist was used as the reference marker for pattern fill. Rigid-body fill was used primarily for missing head or trunk markers. Marker dropout occurred mainly around the wrist and the jugular notch and xiphoid process markers. Exact gap lengths for individual trials were not retrospectively reconstructed (see Limitations). No digital filtering or smoothing was applied to the marker trajectories.

In the exported C3D files used here, any residual frame in which a marker required for a given outcome was still unavailable was removed by listwise deletion before that outcome was computed; across the 18 analyzed trials this affected 0-9.3% of frames per trial (mean 0.6%), with only two trials exceeding 1% (four-note slurred scale in P3, 9.3%; spiccato scale in P2, 1.5%). Among the trials contributing to the three descriptively nonoverlapping task × outcome combinations, the concerto-excerpt trials had no residual missing frames, and the spiccato trials had none except P2 (1.5%) and A3 (0.4%). The anterior superior iliac spine markers, from which the pelvic-center measures were derived, were less frequently affected by dropout than the wrist markers that feed the elbow and wrist proxy angles and wrist path length; the concerto wrist proxy-angle finding should be interpreted with this in mind. For wrist path length, cumulative frame-to-frame displacement was summed over the retained frames.

Each analyzed trial was a single continuous motion-capture recording lasting 15.1-30.7 seconds (four-note slurred scale, 17.1-22.8 seconds; spiccato scale, 15.1-20.0 seconds; concerto excerpt, 24.1-30.7 seconds). The analysis window for every outcome was the full captured trial (first to last frame of the C3D file); trials were not trimmed to musical onset or offset. Because a single optical motion-capture system recorded all markers, all marker channels were inherently synchronized at the 100 Hz sampling rate, and no multi-device synchronization was required; analog devices such as force platforms were not used in the present analysis. The analysis code and the derived participant-level data tables are available from the corresponding author on reasonable request (see Acknowledgements); the expanded Methods provide substantially greater detail to support reproducibility.

No participant-specific test-retest assessment or minimal detectable change (MDC) was established for the kinematic outcomes in this study. Small between-group differences in pelvic-center displacement variability were therefore not treated as established separations; in particular, the 0.15-mm separation in vertical pelvic-center displacement variability during spiccato cannot be distinguished confidently from measurement uncertainty and should not be interpreted as a meaningful between-group difference (see Results and Discussion).

Descriptive statistical analysis

Because the sample size was small (three professional and three amateur performers), the primary analysis was descriptive and hypothesis-generating, and no inferential statistical tests were performed. For each of the 10 kinematic outcomes across the three analyzed performance tasks, individual participant values were examined separately, yielding 30 task × outcome combinations in total.

The study was not prospectively registered. The set of kinematic outcomes, the descriptive nonoverlap criterion, and the decision to restrict the analysis to the three tasks with complete data from all six participants were all defined after data collection. To make the selection logic explicit, the 10 outcomes are the complete set derivable from the marker model used (mean and SD of three upper-limb proxy angles, six outcomes; SD of pelvic-center position in three axes, three outcomes; wrist path length, one outcome), and no outcome was computed and then discarded. All tasks with complete data from all six participants were analyzed; the one-note-per-bow scale was excluded only because one professional participant (P3) had no usable data for it. This complete-case exclusion was applied before the descriptive comparison and affected both groups equally. All 30 combinations were assessed; the three nonoverlapping combinations are presented individually, and the overlapping status of the remaining 27 is reported in the text. Because the outcome set and the screening criterion were specified after data collection, the between-group comparison is reported as a post hoc, exploratory screen rather than as a prespecified hypothesis test.

For each task × outcome combination, we assessed whether the range of values observed in the three professional performers overlapped with the range observed in the three amateur performers. A combination was described as showing a “descriptively nonoverlapping pattern” when all three professional values were either greater than all three amateur values or lower than all three amateur values. This designation was used solely as a descriptive criterion and was not interpreted as evidence of statistical significance or a population-level group effect (see the chance calculation below).

With three participants per group, the probability that all three professional values fall entirely above, or entirely below, all three amateur values for a given task × outcome combination is 2 / C(6,3) = 0.10 under a null model of exchangeable participants. Across the 30 combinations examined, the expected number of descriptively nonoverlapping patterns under this null model is therefore approximately 3.0. This expected value is used as the reference against which the observed count is interpreted. The descriptive nonoverlap criterion is not a significance test, and a nonoverlapping pattern confined to a single task was not treated as evidence of an expertise-related difference.

To characterize individual heterogeneity across tasks, task-averaged values were also calculated for each participant by averaging each kinematic outcome across the three analyzed tasks. These participant-level summaries were used to illustrate within-group variation and were not used for inferential group comparisons. The consistency of any nonoverlapping pattern across the three performance tasks was also examined descriptively.

Ethical considerations

The study protocol was reviewed and approved by the Research Ethics Committee for Health and Medical Sciences at Bunkyo Gakuin University on September 14, 2023 (approval no. 2023-0007), before data collection. All six participants provided written informed consent before participation. The study was conducted in accordance with the approved protocol and applicable ethical standards for research involving human participants.

## Results

Individual heterogeneity within expertise groups

Participant-specific values averaged across the three analyzed tasks are shown in Table 2. Substantial interindividual heterogeneity was observed within both expertise groups across several kinematic outcomes. Among the professional performers, task-averaged mediolateral pelvic-center displacement variability ranged from 8.88 mm in P1 to 83.64 mm in P3, representing an approximately 9.4-fold difference. Within the amateur group, the task-averaged mean right shoulder geometric proxy angle ranged from 123.42° in A3 to 143.39° in A1, a difference of approximately 20°. Similar overlap between professional and amateur participant values was observed for most of the remaining task-averaged outcomes.

**Table 2:** Participant-specific kinematic values averaged across the three analyzed tasks. Values are participant-specific means across the four-note slurred scale, spiccato scale, and concerto excerpt. “Variability” in the proxy-angle SD and pelvic-center rows denotes within-trial temporal variability. Wrist path length is the raw cumulative value; per-second values (not shown) yield the same nonoverlap screening result. Ama, amateur performer; Pro, professional performer

Metric	P1 (Pro)	P2 (Pro)	P3 (Pro)	A1 (Ama)	A2 (Ama)	A3 (Ama)
Right shoulder geometric proxy angle, mean (deg)	127.95	131.05	121.94	143.39	130.28	123.42
Right shoulder geometric proxy angle, SD (deg)	5.48	7.29	8.69	9.49	4.41	9.24
Right elbow geometric proxy angle, mean (deg)	103.79	91.59	92.93	96.04	92.4	80.07
Right elbow geometric proxy angle, SD (deg)	19.9	12.3	17.35	18.69	13.71	15.47
Right wrist geometric proxy angle, mean (deg)	138.25	155.02	155.82	135.39	143.54	156.79
Right wrist geometric proxy angle, SD (deg)	9.88	10.34	7.77	11.61	10.39	12.34
Pelvic-center displacement variability, mediolateral (mm)	8.88	51.01	83.64	34.85	7.64	11.95
Pelvic-center displacement variability, anteroposterior (mm)	21.32	16.54	21.05	15.53	5.87	7.77
Pelvic-center displacement variability, vertical (mm)	1.59	4.12	3.93	1.9	0.88	1.79
Wrist path length (mm)	7943.47	7336.96	8309.73	7285.13	5251.46	8186.08

Within-group heterogeneity was not limited to a single kinematic domain. Among the professional performers, task-averaged wrist path length ranged from 7,336.96 mm in P2 to 8,309.73 mm in P3, while mean right wrist geometric proxy angle ranged from 138.25° in P1 to 155.82° in P3. Among the amateur performers, task-averaged wrist path length ranged from 5,251.46 mm in A2 to 8,186.08 mm in A3, and mean right elbow geometric proxy angle ranged from 80.07° in A3 to 96.04° in A1. These participant-level distributions showed that substantial heterogeneity was present across both upper-limb and pelvic-motion measures within each expertise category.

Overall, the task-averaged participant profiles showed substantial overlap between the professional and amateur groups across all 10 kinematic outcomes. No task-averaged measure showed complete separation between all three professional and all three amateur performers. Task-specific values were therefore examined separately to identify whether any descriptively nonoverlapping patterns occurred within individual performance tasks.

Kinematic measures showing a descriptively nonoverlapping pattern between groups

Across the 30 task × outcome combinations, three showed descriptively nonoverlapping value ranges between the professional and amateur performers. During the spiccato task, anteroposterior pelvic-center displacement variability was higher in all three professionals than in all three amateurs (professional range, 15.29-26.63 mm; amateur range, 4.73-13.02 mm). Vertical pelvic-center displacement variability during spiccato showed the same directional pattern (professional range, 1.78-3.04 mm; amateur range, 0.81-1.63 mm). During the concerto excerpt, right wrist geometric proxy angle variability was lower in all three professionals than in all three amateurs (professional range, 11.11-15.01°; amateur range, 16.01-18.05°). The remaining 27 task × outcome combinations showed overlapping value ranges between the two expertise groups.

Individual participant values for the three descriptively nonoverlapping task × outcome combinations are presented in Table 3. For both spiccato pelvic-center displacement measures, the lowest professional value remained higher than the highest amateur value. Conversely, for right wrist geometric proxy angle variability during the concerto excerpt, the highest professional value remained lower than the lowest amateur value. No additional task × outcome combination met this complete nonoverlap criterion.

**Table 3:** Participant-level values for task × outcome combinations showing descriptively nonoverlapping patterns. “Descriptively nonoverlapping” indicates complete separation of the observed ranges and does not imply statistical significance or a population-level group effect. Three of 30 combinations met this criterion, consistent with the number expected by chance (≈ 3); see text. Study-specific measurement precision was not established, so the vertical pelvic-center separation (1.78 vs 1.63 mm) cannot be interpreted as meaningful. A1-A3, amateur performers; P1-P3, professional performers

Task	Metric	P1	P2	P3	A1	A2	A3
Spiccato scale	Pelvic-center displacement variability, anteroposterior (mm)	26.63	15.29	16.08	13.02	4.73	7.16
Spiccato scale	Pelvic-center displacement variability, vertical (mm)	1.78	3.04	2.89	1.63	0.81	1.13
Concerto excerpt	Right wrist geometric proxy angle, SD (deg)	15.01	11.25	11.11	16.08	16.01	18.05

This observed count (three of 30) matches the number expected by chance alone under a null model of exchangeable participants (expected ≈ 3.0; see Materials and Methods). None of the three patterns recurred across tasks, providing no cross-task corroboration of an expertise-related effect. The three combinations are therefore reported as descriptive observations and candidate hypotheses, not as evidence of expertise-related differences.

For vertical pelvic-center displacement variability during spiccato, no participant-specific test-retest assessment or MDC was established; the 0.15-mm separation between the lowest professional value (1.78 mm) and the highest amateur value (1.63 mm) therefore cannot be distinguished confidently from measurement uncertainty and should not be interpreted as a meaningful between-group difference. The anteroposterior pelvic-center difference during spiccato (professional range 15.29-26.63 mm vs amateur range 4.73-13.02 mm) is larger in absolute and relative terms but, as noted above, remains within the number of nonoverlapping patterns expected by chance.

Task dependence of the observed patterns

The descriptively nonoverlapping patterns were task-specific. The two pelvic-center displacement variability measures were observed only during the spiccato task, whereas the right wrist geometric proxy angle variability pattern was observed only during the concerto excerpt. No kinematic outcome showed descriptively nonoverlapping professional and amateur ranges in more than one of the three analyzed tasks, and no such pattern was identified during the four-note slurred scale.

Expressing wrist path length per second of the analyzed recording, rather than as a raw cumulative distance, did not change which combinations met the nonoverlap criterion.

Taken together, the descriptive analysis showed substantial interindividual heterogeneity across the measured kinematic outcomes, with broad overlap between professional and amateur performers for most task × outcome combinations. Complete nonoverlap between expertise groups was limited to three of 30 combinations and was restricted to specific performance tasks. No kinematic outcome showed descriptively nonoverlapping professional and amateur ranges across multiple tasks.

## Discussion

The principal finding of this pilot study was the substantial interindividual heterogeneity and task-specific kinematic patterns observed in violin playing. Of the 30 task × outcome combinations examined, only three showed descriptively nonoverlapping ranges between professional and amateur performers, and no such pattern occurred in more than one task. These consisted of greater anteroposterior and vertical pelvic-center displacement variability among the professional performers during spiccato and greater right wrist geometric proxy angle variability among the amateur performers during the concerto excerpt. Most other measures showed substantial overlap between the two expertise groups. Thus, the present descriptive findings provide limited evidence for a kinematic pattern that consistently characterizes professional or amateur violinists across different performance tasks.

The number of descriptively nonoverlapping task × outcome combinations (three of 30) is equal to the number expected by chance alone with three participants per group (expected ≈ 3.0), and none of the three combinations recurred across tasks. These findings should therefore be read as chance-consistent descriptive observations rather than as evidence of expertise-related kinematic differences; the more robust observation is the substantial interindividual heterogeneity present within each expertise group.

Previous studies have demonstrated that violin-playing kinematics can contain features associated with expertise level, including motion-capture characteristics that distinguish professional from less-skilled performers [7]. The present findings do not contradict such group-level observations; rather, they indicate that expertise-related differences may coexist with substantial variability among performers within the same expertise category. This interpretation is consistent with prior work showing appreciable intra- and intermusician variability in violin joint kinematics across bow strokes, tempi, and strings [5]. Similarly, studies of repetitive violin bowing have shown that coordination patterns depend on performance conditions and may differ substantially across individual players [8]. In the present pilot sample, the broad overlap between professional and amateur values for most outcomes therefore supports participant-level and task-specific reporting as a complement to group-level summaries, particularly when only a small number of performers are available.

Because the three nonoverlapping combinations are consistent with chance and point in opposite directions (greater pelvic displacement variability in professionals during spiccato; lower wrist proxy-angle temporal variability in professionals during the concerto), we do not offer a unifying physiological account. We note only that an interpretation broad enough to treat either direction as “consistent with expertise” would not be constrained by these data. If, in an adequately powered and prespecified study, professionals were confirmed to show greater axial-body displacement variability during rapid off-string bowing, one candidate interpretation would be that pelvic motion forms part of a distributed whole-body coordination strategy rather than reduced postural control [1,5,9]; conversely, if professionals were confirmed to show lower wrist proxy-angle temporal variability during musically complex passages, a candidate interpretation would be a more repeatable distal configuration. Both remain untested hypotheses. Because bow force, sound quality, and muscle activity were not measured, the functional meaning of any such task-specific difference cannot be established here.

The substantial interindividual heterogeneity observed in this pilot study may also be relevant to performing-arts medicine and rehabilitation. PRMDs are common among violinists and other instrumental musicians, but their anatomical distribution, severity, and functional consequences vary across performers [3,10]. Reviews of violin and viola performance have likewise emphasized that musculoskeletal loading reflects a combination of posture, muscle activity, joint mobility, instrument-related factors, and individual characteristics rather than a single uniform mechanism [11]. From a rehabilitation perspective, this heterogeneity supports approaches that consider the individual musician, instrument, task demands, symptoms, and functional goals rather than applying a single movement model to all performers [12]. Current evidence for strategies to prevent and manage musicians’ musculoskeletal symptoms remains limited and heterogeneous, highlighting the need for further clinically oriented research [13]. Because this study collected no symptom, pain, disability, or injury data, it cannot address musculoskeletal risk directly; its role is limited to motivating future studies that would combine individualized kinematic profiling with prospective symptom and functional outcome measurement.

Limitations

Several limitations should be considered when interpreting these findings. First, the sample was very small (n = 6; three performers per expertise group), and the professional and amateur participants were not matched for age, sex, years of violin experience, or weekly practice time: professionals were 33-41 years old with 29-36 years of playing experience, whereas amateurs were 21-57 years old with 6-38 years of experience, and one amateur reported more years of playing than any professional. Years of experience is therefore not a clean proxy for expertise in this sample, and the observed descriptive patterns cannot be attributed to professional status independent of age and experience. Second, only the single analysis trial recorded after the practice performance was analyzed for each participant and task, so trial-to-trial reliability and within-performer variability could not be assessed. Third, one professional participant lacked usable data for the one-note-per-bow task, requiring the primary analysis to be restricted to the three tasks with complete data across all six performers. Fourth, the upper-limb measures were three-point geometric proxy angles rather than anatomically defined 3D joint angles, and no concurrent measures of bow force, sound quality, muscle activity, pain, fatigue, or playing-related musculoskeletal symptoms were available. Fifth, marker gaps were filled in Vicon Nexus (spline, pattern, and rigid-body fill) before analysis, and the exact gap lengths per trial were not retrospectively reconstructed; gap-filled segments, particularly longer pattern- or rigid-body-filled segments around the wrist and the anterior thorax, may not exactly reproduce true marker motion, adding uncertainty to the SD-based (within-trial temporal-variability) outcomes and to wrist path length. No digital filtering or smoothing was applied, and this may increase measurement noise in SD-based outcomes. No participant-specific test-retest assessment or MDC was established, so small between-group differences in pelvic-center displacement variability, including the 0.15-mm vertical pelvic-center difference during spiccato, cannot be distinguished confidently from measurement uncertainty. Sixth, the standardized recording phase always followed the familiarization/warm-up phase, so order-related effects such as adaptation or fatigue could not be separated from the standardized recording condition. Finally, the study was not preregistered; the outcome set, the nonoverlap criterion, and the task-inclusion decision were defined after data collection, and the analysis should be regarded as an exploratory screen despite the explicit reporting of the full selection logic. Future studies should include larger and more diverse samples, repeated trials, validated 3D joint kinematics, synchronized performance measures, and clinically relevant symptom assessments. Longitudinal or repeated-measures designs may also help determine whether performer-specific kinematic profiles are stable over time and whether they are associated with musculoskeletal symptoms, functional limitations, or responses to individualized rehabilitation.

## Conclusions

Violin-playing kinematics showed substantial interindividual heterogeneity and task-specific kinematic patterns in this small pilot cohort. Descriptively nonoverlapping professional-amateur ranges were limited to three of 30 task × outcome combinations, the number expected by chance. None recurred across tasks, so this pilot provides no evidence for a kinematic profile that consistently distinguishes professional from amateur performers. Because of the small, demographically heterogeneous sample and the descriptive, non-preregistered design, the findings are hypothesis-generating. Future adequately powered studies integrating repeated kinematic assessment with prospectively measured playing-related musculoskeletal symptoms and functional outcomes would be needed to determine whether individualized movement profiling has practical value for performer-specific assessment, prevention, or rehabilitation.
